# Psychometric Properties of the Mandarin Version of the Quality Improvement Self‐Efficacy Inventory Among the Nurses: A Methodological Study

**DOI:** 10.1155/jonm/4425503

**Published:** 2026-01-19

**Authors:** Nuoyan Zhang, Xiaoxiao Zhang, Zhaonan Wang, Yuting Feng, Hangjie Lian, Haoquan Han, Xinyu Bai, Yaqi Huang, Yulu Wang, Yue Zhao, Qi Lu

**Affiliations:** ^1^ Intelligent Nursing Research Center, Tianjin Medical University, Tianjin, China, tijmu.edu.cn; ^2^ School of Nursing, Tianjin Medical University, Tianjin, China, tijmu.edu.cn; ^3^ Tianjin Medical University General Hospital, Tianjin, China, tjmugh.com.cn; ^4^ School of Nursing, The Hong Kong Polytechnic University, Hong Kong, China, polyu.edu.hk; ^5^ Joint Research Centre for Primary Health Care, The Hong Kong Polytechnic University, Hong Kong, China, polyu.edu.hk

**Keywords:** nurse, power analysis, psychometric properties, Quality Improvement Self-Efficacy Inventory

## Abstract

**Background:**

Quality improvement is crucial for improving healthcare systems and providing high‐quality, safe care for patients. Accurate assessment of nurses’ quality improvement knowledge and skills is necessary to integrate quality improvement into nursing practice. The Quality Improvement Self‐Efficacy Inventory measures nurses’ knowledge and skills in quality improvement.

**Objectives:**

To translate the Quality Improvement Self‐Efficacy Inventory into Mandarin and evaluate its psychometric properties among nurses.

**Design:**

A Methodological Study.

**Setting:**

At a tertiary hospital in Tianjin, China, between January and April 2023.

**Participants:**

A total of 436 nurses participated in the psychometric evaluation, and 9 nurses participated in the cognitive interviews.

**Methods:**

The Quality Improvement Self‐Efficacy Inventory was translated into Mandarin using Brislin’s translation model. Item analysis was conducted using extreme group, correlation coefficient, corrected item‐total correlation and Cronbach’s alpha coefficient methods. Both exploratory and confirmatory factor analyses were employed to determine the factor structure and evaluate the proposed model. A likelihood ratio test and power analysis were combined to test hypotheses regarding the model fit. Structural, convergent and discriminant validity were assessed. Reliability was evaluated using internal consistency and test–retest reliability.

**Results:**

The Mandarin‐Quality Improvement Self‐Efficacy Inventory consists of 10 items, all exhibiting sufficient homogeneous and good discrimination abilities (coefficient of variance = 0.233–0.306; critical ratio = 17.943–25.348; corrected item‐total correlation = 0.769–0.877). Exploratory factor analysis revealed a new two‐factor structure, with a total variation of 74%. Confirmatory factor analysis indicated good model fit indices for the new two‐factor and original four‐factor models. The new two‐factor structure and original four‐factor structure were considered valid based on the results from the likelihood ratio test and power analysis. The composite reliability and average variance extracted indicate good convergent and discriminant validity. Internal consistency (Cronbach’s alpha coefficient = 0.947) and test–retest reliability (intraclass correlation coefficient = 0.807) of the Mandarin‐Quality Improvement Self‐Efficacy Inventory were excellent.

**Conclusions:**

The Mandarin‐Quality Improvement Self‐Efficacy Inventory, encompassing both the new two‐factor structure version (Mandarin‐Quality Improvement Self‐Efficacy Inventory‐2) and the original four‐factor structure version (Mandarin‐Quality Improvement Self‐Efficacy Inventory‐4), proved to be a reliable, effective, stable and relatively concise measurement instrument. The Mandarin‐Quality Improvement Self‐Efficacy Inventory‐2 can serve as a practical instrument to assess Chinese nurses’ quality improvement knowledge and skills. The Mandarin‐Quality Improvement Self‐Efficacy Inventory‐4 can be used to investigate cross‐cultural measurement invariance in future studies. Two recommendations were proposed as a methodological reference for factor structure research in the development, translation and psychometric evaluation of measurement instruments.

## 1. Background

Ensuring high‐quality and safe patient care is an important global concern. Quality improvement is the systematic and continuous process aimed at raising service standards and addressing healthcare system issues, thereby leading to improved patient outcomes [1]. The implementation of quality improvement can mitigate medical errors and failures, improve the quality of patient care and reduce morbidity and mortality [2]. Nurses play a crucial role in providing safe and efficient patient care in healthcare systems. They need to be proficient in assessing scientific evidence to discern quality care criteria, identify gaps in current care, implement measures to rectify the identified gaps and effectively provide high‐quality and safe patient care. Thus, it becomes imperative to ascertain the extent of nurses’ knowledge and skills in quality improvement practice. However, a global investigation of nurses’ quality improvement competencies indicates a pervasive lack of adequate learning opportunities for their development; many are ill‐prepared or unfamiliar with the quality improvement concept through academic programs [3]. Patients rely on nurses to provide high‐quality and safe care; hence, deficiencies in nurses’ quality improvement knowledge and skills may impede substantial improvement in patient care [4]. Therefore, to effectively integrate quality improvement into nursing practice and ensure the delivery of safe, high‐quality patient care, accurately assessing nurses’ knowledge and skills in quality improvement is crucial. This assessment contributes to the implementation of targeted quality improvement initiatives while generating actionable insights for nursing managers. In the long term, these insights can guide the development of evidence‐based training programs, optimise resource allocation and support the professional growth of nursing staff. This enables them to address evolving healthcare challenges and advance quality improvement efforts, ultimately laying a strong foundation for sustained improvements in both nursing practice and patient care outcomes.

The total number of registered nurses in China had exceeded 5.6 million by 2023. Therefore, there is an urgent need to develop targeted and effective nursing measures to enhance the quality of care and promote continuous professional development. However, the absence of validated, reliable or acceptable measurement instruments for assessing quality improvement implementation among Chinese nurses has resulted in ambiguity regarding nurses’ role in quality improvement in China [5]. The Quality Improvement Self‐Efficacy Inventory efficiently assesses nurses’ quality improvement competencies and skills through streamlining and operability. It was developed by Baernholdt et al. in the United States in 2022, based on a clinical learning environment framework created by the National Collaborative for Improving the Clinical Learning Environment. In a cross‐sectional study involving a convenience sample of 886 nurses in Alabama, the Quality Improvement Self‐Efficacy Inventory was first used to evaluate staff nurses’ quality improvement knowledge and skills. Introducing the Quality Improvement Self‐Efficacy Inventory to China can provide an appropriate tool for evaluating nurses’ quality improvement competencies, thereby contributing to the advancement of nurses’ professional skills to optimise care quality.

While validating the translated version of scales, researchers often prioritise exploring an optimal factor structure rather than replicating the original one. This approach frequently results in discrepancies between the translated and original factor structures, often leading to the abandonment of the original. Such inconsistencies pose significant challenges to the international applicability and cross‐cultural comparability of these instruments. Moreover, in psychometric evaluation, ensuring the validity of factor structures, particularly any newly derived ones, remains a significant challenge. Conventional validation methods predominantly rely on fit indices to assess overall model fit. While these indices effectively measure statistical goodness of fit, they do not account for the risk of Type II error (*β* error) in factor structure decision‐making. Neglecting β errors increases the likelihood that observed adjustments fail to reflect true underlying factor structure effects, instead arising from sampling variability or random error, thereby increasing the likelihood of supporting spurious or misspecified structures that lack substantive meaning.

To address the aforementioned issues, this study translated the Quality Improvement Self‐Efficacy Inventory into Mandarin and evaluated its psychometric properties among the Chinese nursing population. Additionally, by incorporating power analysis to evaluate whether the original and translated factor structures could detect true underlying effects, the study aims to provide a methodological reference for future research in the development, translation and psychometric evaluation of measurement instruments.

## 2. Methods

### 2.1. Study Design

This study included cognitive interviews and a psychometric evaluation of the Quality Improvement Self‐Efficacy Inventory. The study was conducted in two phases. In Phase I, the Quality Improvement Self‐Efficacy Inventory was translated into Mandarin. Phase II involved the evaluation of the psychometric properties of the Mandarin‐Quality Improvement Self‐Efficacy Inventory within a nurse population.

### 2.2. Phase I: Translation Procedures

#### 2.2.1. Forward and Back Translation

With authorisation from Professor Marianne Baernholdt, the original Quality Improvement Self‐Efficacy Inventory was translated into Mandarin, strictly following Brislin’s translation model [6]. A bilingual nursing professor who had completed 5 years of study in the United States translated the scale into Mandarin, forming the initial Mandarin‐Quality Improvement Self‐Efficacy Inventory. Subsequently, another bilingual nursing professor with more than 10 years of clinical experience in China, blinded to the original scale, back‐translated the initial Mandarin‐Quality Improvement Self‐Efficacy Inventory into English. A nursing expert well‐versed in medical translation, with a minimum of 5 years of actual translation experience in the medical field, compared the back‐translated version with the original Quality Improvement Self‐Efficacy Inventory item by item and modified controversial items. This expert, together with the full author team, who have backgrounds in nursing management, quality improvement and psychometrics, then served as an expert review panel for the prefinal Mandarin version. The panel examined each item for conceptual relevance to quality improvement self‐efficacy, representativeness of the construct, cultural and contextual appropriateness in Chinese nursing practice, and clarity and comprehensibility of wording. This expert review was used as a qualitative assessment of content validity. Finally, once consensus was reached, the prefinal Mandarin‐Quality Improvement Self‐Efficacy Inventory was established.

#### 2.2.2. Cognitive Interviews

To assess the clarity, applicability and comprehensiveness of the prefinal Mandarin‐Quality Improvement Self‐Efficacy Inventory and to gain further insight into subjects’ interpretations of the items, cognitive interviews were conducted face‐to‐face with a purposive sampling of 9 nurses recruited from the target population. This interview took place in an undisturbed office within the hospital and ranged in duration from 20 to 30 min, using the ‘think‐aloud’ and ‘verbal‐probing’ techniques [7]. The nurses were asked to verbalise their thought processes while responding to each item and were subsequently probed verbally based on Tourangeau’s four‐stage response model [8]. Cognitive interviews did not reveal any issues. Consequently, the final revision of the Mandarin‐Quality Improvement Self‐Efficacy Inventory, with good face validity, was developed and used for further psychometric evaluation.

### 2.3. Phase II: Psychometric Evaluation

#### 2.3.1. Participants

The participants in this study were registered nurses who provided direct clinical care in clinical departments. Inclusion criteria for participants were as follows: (a) have obtained the Chinese Nurse Practitioner Certificate and are currently involved in clinical nursing, (b) have been involved in clinical nursing for over 1 year and (c) provide informed consent and willingness to participate in the study. Exclusion criteria included nurses who were absent from work during the survey period due to vacation, studying abroad, sickness, etc.

The sample size was determined based on three recommendations: (a) 5–10 participants per item to accurately evaluate construct validity and internal consistency [9]; (b) using different subsamples to conduct exploratory factor analysis and confirmatory factor analysis to facilitate a cross‐validation test [10]; (c) a minimum of 200 participants per subsample for exploratory factor analysis and confirmatory factor analysis [11]. Following these three recommendations, a minimum sample size of 400 participants was considered adequate for our study. Assuming 10% of possible missing responses, we aimed to recruit 440 participants.

#### 2.3.2. Instrument

The questionnaire comprised two sections: a demographic questionnaire and the Mandarin version of the Quality Improvement Self‐Efficacy Inventory.

The demographic questionnaire was self‐designed and included questions on sex, age, department, working years, professional titles, education background, post, weekly working hours, marital status, number of children and whether participants had undergone quality improvement training.

The Mandarin version of the Quality Improvement Self‐Efficacy Inventory was translated from the original version in Phase I. The original Quality Improvement Self‐Efficacy Inventory consists of four dimensions with 10 items: alignment with safety culture (two items), recognition and reporting (three items), participation and analysis (three items) and translation and action (two items). It employs a 4‐point Likert scale ranging from 1 to 4 (1 = not confident, 2 = somewhat confident, 3 = confident, 4 = very confident) to gauge nurses’ confidence levels in performing each quality improvement behaviour. The total score ranged from 0 to 40. The overall rating was calculated by averaging the total scores; a higher mean indicates greater confidence in executing each quality improvement behaviour. The Mandarin version was semantically equivalent to the original English version and included 10 items scored identically to the original. A higher average total score reflects greater confidence in executing each quality improvement behaviour by the nurses.

#### 2.3.3. Data Collection

A cross‐sectional observational survey was conducted at a tertiary hospital in Tianjin, China, from January to April 2023. The survey randomly involved 18 selected departments and was conducted during regular departmental nursing meetings. After precalculating the number of participants, 461 nurses who met the inclusion and exclusion criteria were identified as potential study participants.

We used the online survey platform WenjuanXing (https://www.wjx.cn), a Chinese platform that supports customisable surveys with both single‐ and multiple‐choice questions, to create the questionnaire. We generated unique online questionnaire links by incorporating a key parameter, ‘sojumpparm’, which indicates ‘customised link parameters’. A total of 461 distinct parameter values (e.g., N001, N002, etc.) were created and embedded into the links (e.g., sojumpparm = N001). Each parameter value was designed to allow only one response, preventing multiple submissions from the same participant. In total, 461 specified questionnaire links were generated. Each link is unique to an individual participant and restricted to a single response. These links were converted into QR codes, allowing participants to scan and access the questionnaire directly from their devices without needing to manually enter a Uniform Resource Locator (URL), making the survey process more convenient and efficient. Nurses who met the inclusion criteria scanned the printed QR codes and completed the questionnaire onsite, ensuring that the questionnaire could not be circulated outside the hospital for internal quality control purposes.

Finally, 440 (95.4%) of the 461 nurses completed the survey, while 21 (4.6%) were unable to complete the survey as they left the meeting early due to work‐related reasons. Subsequently, within each department, we generated a list of eligible nurses who had completed the baseline survey and were expected to be available at the follow‐up time point. Using a random number table applied to the departmental rosters, we initially selected approximately two to three nurses per department to form a candidate retest group. Because participation in the retest assessment was voluntary and depended on work schedules, some initially selected nurses declined or were unavailable at the two‐week follow‐up, and replacement nurses from the same department were approached. In total, 50 nurses completed the Mandarin‐Quality Improvement Self‐Efficacy Inventory a second time after an interval of two weeks, and their data were used to estimate test–retest reliability.

### 2.4. Statistical Analysis

Descriptive analyses were employed to describe the demographic characteristics of participants.

Item analysis encompassed the extreme group method, correlation coefficient method, corrected item‐total correlation and Cronbach’s alpha coefficient method. Items with a coefficient of variance < 0.15 and a critical ratio < 3 or *p* > 0.05 were excluded. Pearson’s correlation coefficient was used to examine the correlation between the scores for each item and the scale. Items with a correlation coefficient of < 0.3 or a corrected item‐total correlation of < 0.40 were excluded [12], along with items whose original Cronbach’s alpha coefficient was lower than that obtained following the deletion of the item.

Scale validity was assessed through structural validity, convergent validity and discriminant validity. Structural validity was verified using a cross‐validation approach employing both exploratory factor analysis and confirmatory factor analysis. Before conducting the exploratory factor analysis, Bartlett’s sphere test and Kaiser–Meyer–Olkin tests for sampling adequacy were performed to determine the suitability of the exploratory factor analysis [13]. The data were standardised, a correlation matrix was calculated, and the number of factors to be extracted was determined. As the maximum likelihood approach occasionally fails to converge, the iterated principal axis approach was adopted to extract the unrotated factors, followed by oblique rotation (promax rotation) to aid in factor interpretation. Confirmatory factor analysis was performed using maximum likelihood estimation to cross‐validate the factor structure derived from the exploratory factor analysis, and the overall model was evaluated using chi‐square likelihood ratio tests and fit indices. The chi‐square likelihood ratio test evaluates the exact fit, whereas fit indices quantify how well a model fits the data. A good model fit was indicated if [14]: chi‐square/degree of freedom < 3.0; comparative fit index, adjusted goodness‐of‐fit index, goodness‐of‐fit index, normed fit index ≥ 0.95 (or ≥ 0.90 for acceptable fit); Tucker–Lewis index ≥ 0.95 (or ≥ 0.90 for acceptable fit); standardised root mean square residual ≤ 0.08; and root mean square error of approximation ≤ 0.05 (or ≤ 0.08 for reasonable fit) along with 90% confidence intervals. The average variance extracted (AVE) and composite reliability (CR) were utilised to evaluate the convergence validity of the scale, and AVE > 0.5 while CR > 0.7, indicating great convergence validity [15]. Discriminant validity was established if the square root of each factor’s AVE was greater than the correlation coefficient with the other factors [15].

Internal consistency and test–retest reliability were used to gauge scale reliability. Cronbach’s alpha coefficient was adopted to assess internal consistency, and a value of 0.70 or above was commonly considered good internal consistency [16]. Test–retest reliability was evaluated by calculating intraclass correlation coefficients. An intraclass correlation coefficient > 0.75 indicated good repeatability [17].

Since the study involved hypothesis testing (chi‐square likelihood ratio tests), a power analysis was conducted to evaluate the statistical capacity of the model to detect true effects, thereby minimising the risk of supporting a spurious structure. Ideal power was considered to be greater than 80%.

All statistical analyses were conducted using R Version 4.3.3, with the following packages: gplots (Version 3.1.3.1), gtsummary (Version 1.7.2), irr (Version 0.84.1), lavaan (Version 0.6–17), psych (Version 2.4.3), readxl (Version 1.4.3), RColorBrewer (Version 1.1–3), rstatix (Version 0.7.2), semPower (Version 2.1.0), semTools (Version 0.5–6) and tidyverse (Version 2.0.0). The *p* values were two‐sided, with a significance level of 0.05.

### 2.5. Ethical Consideration

This study was reviewed and approved by the ethics committee of Tianjin Medical University (TMUhMEC2022025). Before completing the informed consent form, all the participants received a comprehensive explanation of the study, including its potential risks and benefits. Participants’ right to privacy and anonymity were fully protected. All participants were informed that their data would remain confidential.

## 3. Results

### 3.1. Demographics

A total of 440 nurses completed the survey, of whom four had missing Mandarin‐Quality Improvement Self‐Efficacy Inventory values. The available dataset excluded these four nurses instead of imputing missing values. Ultimately, a total sample size of 436 nurses was included in this study. Details of demographic characteristics are presented in Table 1.

**Table 1:** Demographic characteristics of participants.

Variable	Descriptor	Total sample (*n* = 436)	Subsample 1 (*n* = 218)	Subsample 2 (*n* = 218)
		*n* (%)^∗^		
Sex	Male	16 (3.7)	8 (3.7)	8 (3.7)
	Female	420 (96.3)	210 (96.3)	210 (96.3)

Age	< 30	128 (29.4)	63 (28.9)	65 (29.8)
	30–40	248 (56.9)	122 (56.0)	126 (57.8)
	41–50	45 (10.3)	25 (11.5)	20 (9.2)
	> 50	15 (3.4)	8 (3.7)	7 (3.2)

Working years	< 10	196 (45.0)	96 (44.0)	100 (45.9)
	10–20	202 (46.3)	102 (46.8)	100 (45.9)
	21–30	21 (4.8)	11 (5.0)	10 (4.6)
	> 30	17 (3.9)	9 (4.1)	8 (3.7)

Professional titles	Nurse	59 (13.5)	28 (12.8)	31 (14.2)
	Junior nurse	147 (33.7)	78 (35.8)	69 (31.7)
	Supervisor nurse	222 (50.9)	108 (49.5)	114 (52.3)
	Assistant Director nurse	7 (1.6)	3 (1.4)	4 (1.8)
	Director nurse	1 (0.2)	1 (0.5)	0 (0.0)

Education background	Associate degree	58 (13.3)	31 (14.2)	27 (12.4)
	Bachelor degree	366 (83.9)	179 (82.1)	187 (85.8)
	Master degree	12 (2.8)	8 (3.7)	4 (1.8)
	Doctoral degree	0 (0.0)	0 (0.0)	0 (0.0)

Post	Manage post	34 (7.8)	16 (7.3)	18 (8.3)
	Nonmanagerial post	402 (92.2)	202 (92.7)	200 (91.7)

Weekly working hours	< 40	27 (6.2)	15 (6.9)	12 (5.5)
	40–50	376 (86.2)	186 (85.3)	190 (87.2)
	> 50	33 (7.6)	17 (7.8)	16 (7.3)

Marital status	Married	320 (73.4)	159 (72.9)	161 (73.9)
	Unmarried/Divorced/Bereaved	116 (26.6)	59 (27.1)	57 (26.1)

Number of children	0	141 (32.3)	74 (33.9)	67 (30.7)
	1	217 (49.8)	106 (48.6)	111 (50.9)
	2	75 (17.2)	38 (17.4)	37 (17.0)
	≥ 3	3 (0.7)	0 (0.0)	3 (1.4)

Have received QI training	Yes	160 (36.7)	73 (33.5)	87 (39.9)
	No	276 (63.3)	145 (66.5)	131 (60.1)

^∗^Due to rounding, the percentages might not add up to exactly 100%.

The total sample (*n* = 436) was evenly divided into two adequately sized subsamples according to the random number table method to perform a cross‐validation test for structural validity. The characteristics of both subsamples, as presented in Table 1, closely resembled those of the total sample, with no significant differences observed between the two subsamples.

In addition, the demographic characteristics of the retest subgroup (*n* = 50) were broadly similar to those of the full sample in terms of sex, age, years of nursing experience, education level and department type.

### 3.2. Item Analysis

#### 3.2.1. Item Discrimination

By ranking the total scores in descending order, the top and bottom 27% were assigned to the high‐ and low‐scoring groups. An independent samples *t*‐test was conducted on each item score between the high‐ and low‐scoring groups, and the differences were statistically significant (*p* < 0.001), with the critical ratio ranging from 17.943 to 25.348. The coefficient of variation for each item ranged from 0.233 to 0.306. These results verified that the discrimination ability of the scale item was good, and no item was suggested for removal (Table 2).

**Table 2:** Item homogeneity and discrimination of each term of the Mandarin‐Quality Improvement Self‐Efficacy Inventory.

Item	Coefficient of variance	Critical ratio	Correlation coefficient of item‐scale	Corrected item‐total correlation	Cronbach’s α coefficient if the item is deleted
1. Identifying system issues that contribute to patient safety problems	0.283	17.943^∗^	0.782^∗^	0.726	0.944
2. Applying lessons learnt from mistakes of peers, teams and self to improve patient safety	0.234	18.713^∗^	0.769^∗^	0.717	0.944
3. Communicating concerns about hazards to patients and families	0.233	20.395^∗^	0.777^∗^	0.726	0.944
4. Communicating concerns about hazards to colleagues (team)	0.236	22.069^∗^	0.801^∗^	0.754	0.943
5. Using organisational error reporting systems for near‐miss and error reporting	0.254	25.348^∗^	0.877^∗^	0.846	0.939
6. Engaging in root‐cause analyses when errors or near‐misses occur	0.288	25.049^∗^	0.841^∗^	0.797	0.941
7. Applying tools and methods systematically to collect and analyse data for performance improvement	0.306	23.100^∗^	0.847^∗^	0.803	0.940
8. Working in a team to improve processes or systems of care as a result of errors that were reported back to your unit	0.299	21.802^∗^	0.851^∗^	0.809	0.940
9. Using national patient safety resources, initiatives or regulations such as the National Quality Forum or the Institute of Healthcare Improvement to guide improvement initiatives on your unit	0.289	21.239^∗^	0.847^∗^	0.805	0.940
10. Repeating measurement, assessment and applications of tools for improvement and evaluating changes until the desired performance is sustained	0.300	21.374^∗^	0.839^∗^	0.795	0.941

^∗^ *p* < 0.001.

#### 3.2.2. Homogeneity Test

The correlation coefficients between the score of each item and the scale ranged from 0.769 to 0.877 (*p* < 0.001), and the corrected item‐total correlation values exceeded 0.40, indicating that all items were highly relevant to the scale. The scale’s overall Cronbach’s alpha coefficient was 0.947, which decreased after exclusively deleting any single item. Therefore, no items were deemed necessary for exclusion from the analysis (Table 2).

### 3.3. Validity

#### 3.3.1. Structural Validity

Using the random number table method, the sample (*n* = 436) was evenly categorised into two adequately sized subsamples for a cross‐validation test. The first subsample (*n* = 218) was used for exploratory factor analysis, and the second subsample (*n* = 218) was used for confirmatory factor analysis.

##### 3.3.1.1. Exploratory Factor Analysis

The result of the Kaiser–Meyer–Olkin tests was 0.930, and Bartlett’s sphere test of sphericity was significant (*χ* ^2^ = 2043.298, df = 45, *p* < 0.001), which indicated eligibility to perform exploratory factor analysis. Figure 1(a) shows the results of the factor analysis. According to the Cattell scree test, Horn’s parallel analysis (bootstrap = 100) and the Kaiser–Harris criterion, the factor analysis suggested that two factors fit for extraction. Based on the iterated principal axis approach, two factors were extracted from 10 items, accounting for 74% of the total variation. Performed the Promax rotation to aid in factor interpretation, and the loads of each item on the factors to which they belonged ranged from 0.50 to 0.98, higher than 0.45 [18]. Moreover, as there was no cross‐loading, no items were deleted. Figure 1(b) shows the factor loadings (only factor loadings > 0.45 were retained).

Figure 1Results of the exploratory factor analysis. (a) The result of the factor analysis assessing the number of factors to extract. A scree plot (the line with triangles), eigenvalues greater than 0 criteria (green horizontal line) and parallel analysis with 100 simulations (dashed red line) suggested extracting two factors. (b) Visualisation of result in the oblique two‐factor solution for the Mandarin version of the Quality Improvement Self‐Efficacy Inventory.

**Figure:**
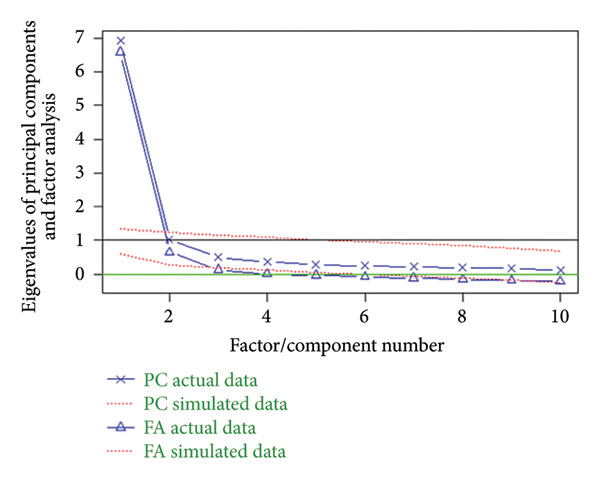
(a)

**Figure:**
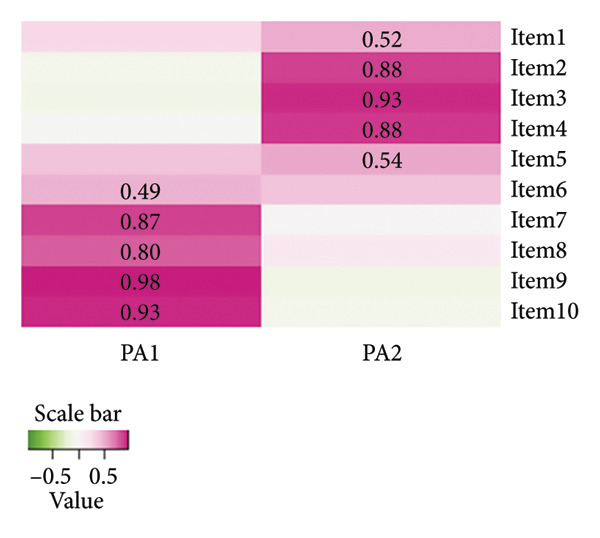
(b)

The factor structure of the Mandarin‐Quality Improvement Self‐Efficacy Inventory differs from that of the original English version. Ten items were reorganised to form a modified two‐factor solution. The first factor explained 40% of the variance and consisted of 5 items (6, 7, 8, 9 and 10). The second factor explained 34% of the variance and consisted of 5 items (1, 2, 3, 4 and 5).

##### 3.3.1.2. Confirmatory Factor Analysis

The two‐factor structure showed satisfactory model fit indices after allowing several theoretically justifiable correlated residuals suggested by modification indices (MI > 4). Specifically, we allowed correlations between the error terms of Items 2, 3 and 4. Item 2 (‘Applying lessons learnt from mistakes of peers, teams, and self to improve patient safety’), Item 3 (‘Communicating concerns about hazards to patients and families’) and Item 4 (‘Communicating concerns about hazard to colleagues (team)’) all focus on learning from and communicating about patient safety problems, and nurses may tend to consider and respond to these closely related communication behaviours in tandem. In addition, we allowed correlations between the error terms of Items 5, 6 and 7. Item 5 (‘Using organisational error reporting systems for near‐miss and error reporting’), Item 6 (‘Engaging in root‐cause analyses when errors or near‐misses occur’) and Item 7 (‘Applying tools and methods systematically to collect and analyse data for performance improvement’) all refer to successive stages in the same quality improvement process (reporting, analysis and use of improvement tools), which may give rise to shared specific variance beyond the common factor. We also allowed a correlated residual between Items 8 (‘Working in a team to improve processes or systems of care as a result of errors that were reported back to your unit’) and 10 (‘Repeating measurement, assessment and applications of tools for improvement and evaluate changes until desired performance is sustained’), because both items focus on team‐based implementation and sustaining cycles of improvement. Finally, we allowed correlated residuals among Items 1 (‘Identifying system issues that contribute to patient safety problems’), 4 and 9 (‘Using national patient safety resources, initiatives or regulations to guide improvement initiatives on your unit’), as these items all emphasise recognising system‐level safety issues and using organisational or national safety frameworks to guide team discussions and improvement work. These correlated residuals, therefore, capture shared specific variance that is not fully accounted for by the two broad latent factors. All standardised factor loadings were statistically significant (*p* < 0.001), ranging from 0.716 to 0.936. The correlation coefficient between the two latent factors was 0.900 (Figure 2(a)). This high interfactor correlation suggests that the two dimensions represent closely related facets of a broader quality improvement self‐efficacy construct rather than completely independent domains, and it should therefore be interpreted with some caution when considering discriminant validity. Given that the original scale had four factors, we performed a confirmatory factor analysis to validate the four‐factor model (Figure 2(b)). The model fit indices for both the proposed two‐factor model and the original four‐factor model are listed in Table 3.

Figure 2Confirmatory factor analysis of the Mandarin‐Quality Improvement Self‐Efficacy Inventory with two‐factor and four‐factor models. (a) The two‐factor model. (b) The four‐factor model. Note: ^∗∗^ *p* < 0.001.

**Figure:**
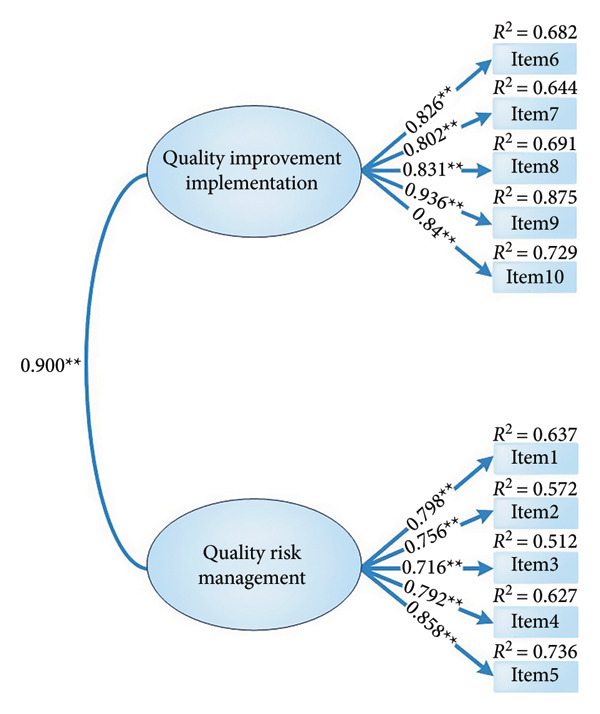
(a)

**Figure:**
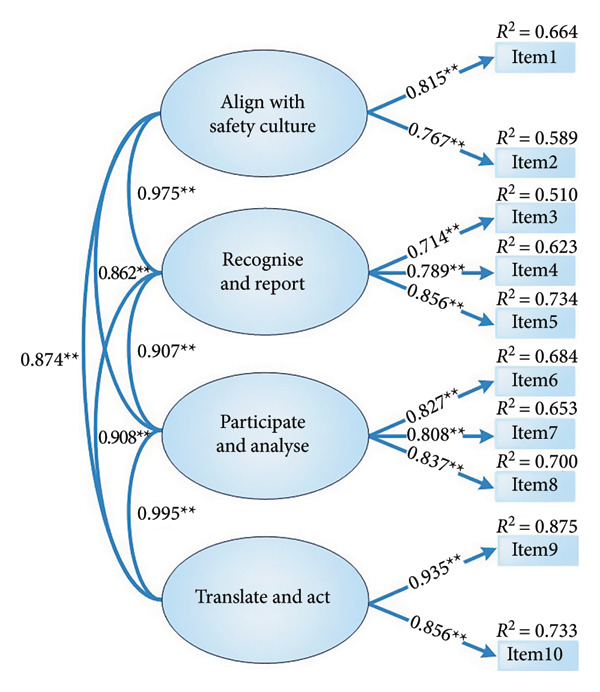
(b)

**Table 3:** Model fit index for the original and proposed models of the Mandarin‐Quality Improvement Self‐Efficacy Inventory (*N* = 218).

Model	*χ* ^2^	*p* value	*χ* ^2^/df	GFI	CFI	AGFI	TLI	NFI	SRMR	RMSEA (CI 90%)
Two‐factor model	37.350	0.053	1.494	0.969	0.994	0.931	0.989	0.982	0.031	0.048 (0.000–0.078)
Four‐factor model	34.571	0.023	1.729	0.971	0.993	0.920	0.984	0.983	0.030	0.058 (0.022–0.090)

*Note:* *χ* ^2^: chi‐square; SRMR: standardised root mean square residual.

Abbreviations: AGFI, adjusted goodness‐of‐fit index; CFI, comparative fit index; df, degree of freedom; GFI, goodness‐of‐fit index; NFI, normed fit index; RMSEA, root mean square error of approximation; TLI, Tucker–Lewis index.

##### 3.3.1.3. *χ* ^2^ Difference Test

The two‐factor model has achieved the categorisation of ‘exact fit’. To provide a more convincing choice for Chinese nurses between our proposed two‐factor model and the original four‐factor model, we conducted the *χ* ^2^ difference test (Δ*χ* ^2^ test). The free parameters in the two‐factor model with fewer restrictions formed a subset of those in the four‐factor model, which had more restrictions. The two models are nested, and the null hypothesis posited that both models would fit equally well. The result of the nested model comparison revealed that the null hypothesis could not be rejected (Δ*χ* ^2^ = 2.778, *p* = 0.734), indicating the four‐factor model could not be considered a better fit. Based on the parsimony principle, the two‐factor model was preferable.

#### 3.3.2. Convergent Validity and Discriminant Validity

In this study, convergent validity was evaluated using CR and AVE, whereas discriminant validity was examined using the Fornell–Larcker criterion and the Heterotrait–Monotrait (HTMT) ratio. As presented in Table 4, the CR and AVE for each factor were greater than 0.7 and 0.5, respectively, and all standardised factor loadings exceeded 0.7. These findings indicate good convergent validity of the scale. The square root of each factor’s AVE was greater than its correlations with the other factor, which supports discriminant validity according to the Fornell–Larcker criterion. The HTMT ratio between the two factors was 0.827, which is below the commonly recommended threshold of 0.85 and therefore supports acceptable discriminant validity [19], although not strongly, between these highly related dimensions.

**Table 4:** Convergent validity and discriminant validity of the Mandarin‐Quality Improvement Self‐Efficacy Inventory.

	CR	AVE	Factor 1	Factor 2
Factor 1	0.937	0.748	0.865^a^	0.827^c^
Factor 2	0.912	0.674	0.799^b^	0.821^a^

Abbreviations: AVE, average variance extracted; CR, composite reliability.

^a^Square root of AVE for each factor.

^b^Correlation coefficient between Factor 1 and Factor 2.

^c^Heterotrait–Monotrait ratio of correlations between Factor 1 and Factor 2.

### 3.4. Reliability

The results revealed that Cronbach’s alpha coefficient was 0.947, indicating that the Mandarin‐Quality Improvement Self‐Efficacy Inventory had excellent internal consistency. The internal consistency estimated for the two dimensions also provided acceptable to excellent reliability, with Cronbach’s alpha coefficients for quality improvement implementation and quality risk management at 0.935 and 0.911, respectively. The Spearman–Brown coefficient of the scale was 0.965, while those of the two dimensions were 0.934 and 0.922, further confirming internal consistency by estimating split‐half reliability (the odd–even trial split). The intraclass correlation coefficient for the scale was 0.807 (95% CI: 0.684–0.886, *p* < 0.001), suggesting high repeatability [17].

### 3.5. Power Analysis

The *p* value of the two‐factor model was above the selected *α* = 0.05 level, indicating that *H*_0_ could not be rejected, suggesting that the model fits the data exactly. Additionally, we performed a power analysis to ensure that the observed nonsignificant test statistic resulted from an accurate model fit rather than a false acceptance of an incorrect model. For the two‐factor model, with a sample size of *N* = 218, the achieved power to detect a small departure from a close fit corresponding to RMSEA = 0.05 at the *α* = 0.05 level was 54% (Figure 3(a)), indicating limited sensitivity to very subtle model misspecifications. By contrast, the power to detect poorer fit corresponding to RMSEA = 0.08 and RMSEA = 0.10 was 96% (Figure 3(b)) and above 99% (Figure 3(c)), respectively, indicating a high probability of identifying clearly misspecified models. Regarding the four‐factor model, we used the compromise power analysis to determine the critical value when forcing the ratio *α*/*β* = 1 (the ratio defines the relative seriousness of both types of errors. Setting the ratio *α*/*β* to 1 implies that neither error is prioritised over the other, and the probabilities of both errors are minimised in a way that reflects their equal perceived consequences), and the *H*_1_ model representing an unacceptable degree of misfit is defined as a model associated with a root mean square error of approximation of at least 0.1. The critical value was 35.714, associated with *α* = *β* ≈ 0.016 (Figure 3(d)). Since the test statistic was smaller than the critical value associated with *α* = *β* ≈ 0.016, the four‐factor model was considered tenable.

Figure 3Results of the power analysis using the central (red solid line) and noncentral (dashed blue line) *χ* ^2^ distribution for null and alternative hypotheses based on root mean square error of approximation. (a) Power to detect misspecifications of a model corresponding to 0.05 on the *α* = 0.05 level. (b) Power to detect misspecifications of a model corresponding to 0.08 on the *α* = 0.05 level. (c) Power to detect misspecifications of a model corresponding to 0.1 on the *α* = 0.05 level. (d) The critical value (dashed black line) obtained from compromise power analysis with forcing the ratio *α*/*β* = 1.

**Figure:**
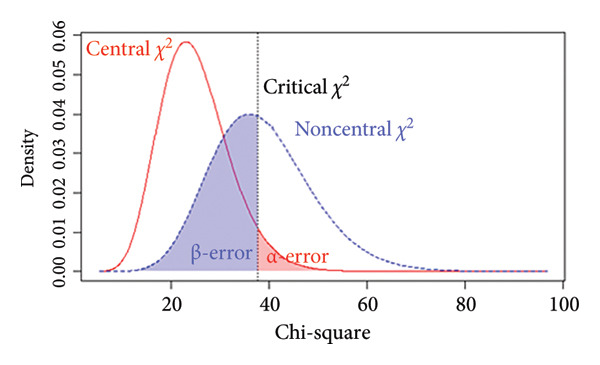
(a)

**Figure:**
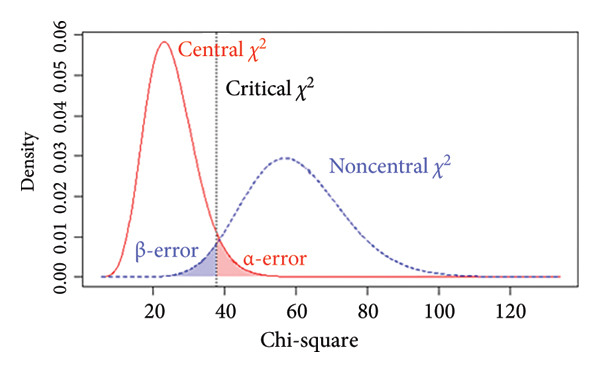
(b)

**Figure:**
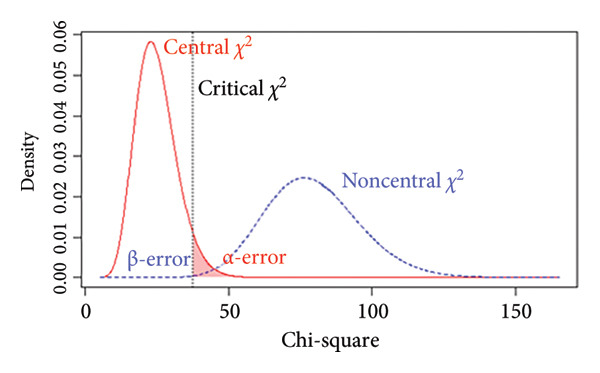
(c)

**Figure:**
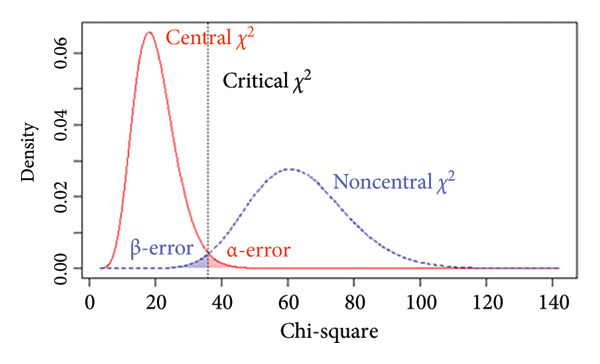
(d)

## 4. Discussion

The Quality Improvement Self‐Efficacy Inventory was translated into Mandarin, and its psychometric properties were evaluated among the Chinese nurses for the first time. The Mandarin‐Quality Improvement Self‐Efficacy Inventory, introducing a new two‐factor structure (Mandarin‐Quality Improvement Self‐Efficacy Inventory‐2), displayed good psychometric properties among Chinese nurses, and the two extracted factors have strong explanatory power. The Mandarin‐Quality Improvement Self‐Efficacy Inventory, retaining the original four‐factor structure (Mandarin‐Quality Improvement Self‐Efficacy Inventory‐4), displayed acceptable psychometric properties. Thus, the Mandarin‐Quality Improvement Self‐Efficacy Inventory, encompassing both the new two‐factor structure version (Mandarin‐Quality Improvement Self‐Efficacy Inventory‐2) and the original four‐factor structure version (Mandarin‐Quality Improvement Self‐Efficacy Inventory‐4), is a reliable, effective, stable and relatively concise measurement instrument. However, because all participants were recruited from a single tertiary hospital in Tianjin, caution is warranted when generalising the findings to nurses working in primary, secondary or rural healthcare settings and to other regions of China where quality improvement resources and organisational cultures may differ. Moreover, although the retest subgroup was initially selected using a random procedure at the department level, the final composition of this group was partly determined by nurses’ availability and willingness to participate in the follow‐up assessment. This may have introduced some selection bias. Accordingly, the test–retest reliability estimate should be interpreted as reflecting stability in a roughly representative, but not strictly probabilistic, subsample of the full sample.

The Mandarin‐Quality Improvement Self‐Efficacy Inventory‐2 is identified with a new two‐factor structure comprising ‘Quality Improvement Implementation’ and ‘Quality Risk Management’, whereas the original scale comprised four factors. We speculate that actual differences in national conditions caused the factor structure discrepancy. The original four‐factor structure was based on a framework created by a national interprofessional task group affiliated with the National Collaborative for Improving the Clinical Learning Environment in the United States. Improvements in nursing quality in China are typically achieved through clinical management and practice [20, 21]. The context should be measured when implementing quality improvement initiatives [5]. Thus, Mandarin‐Quality Improvement Self‐Efficacy Inventory‐2 demonstrates a new two‐factor model (‘quality risk management’ and ‘quality improvement implementation’). Consistent with the conceptual overlap between these domains, the correlation between the two latent factors was high (*r* = 0.900), and the HTMT ratio was 0.827. We therefore interpret ‘Quality Improvement Implementation’ and ‘Quality Risk Management’ as highly related facets of a unified quality improvement self‐efficacy construct rather than completely independent domains. Retaining two factors nonetheless has practical value for nursing managers and educators: distinguishing between risk management‐related behaviours (e.g., hazard identification, communication of concerns and incident reporting) and implementation‐related behaviours (e.g., use of improvement tools, teamwork and sustaining change) may help in designing more targeted training and support strategies, even within a globally coherent construct. Future studies with larger and more diverse samples should examine alternative structural models, such as higher order factor models or bifactor models, to formally test whether a general quality improvement self‐efficacy factor plus specific factors provides a better representation of the construct. In addition, cross‐cultural measurement invariance studies using data from different countries and healthcare systems will be important to determine whether the same factor structure is retained across settings.

Beyond these internal structural indices, however, the present study was not able to provide criterion‐related validity evidence for the Mandarin‐Quality Improvement Self‐Efficacy Inventory. We did not collect data on external psychological constructs or behavioural outcomes that are theoretically related to quality improvement self‐efficacy, such as general self‐efficacy, safety climate, organisational learning climate, participation in quality improvement projects or the frequency of error and near‐miss reporting. Consequently, the validity argument in this study is based mainly on convergent and discriminant validity indices derived from the factor structure (CR, AVE, the Fornell–Larcker criterion and HTMT), and the overall evidence remains partial. Future studies should therefore include measures of related psychological constructs (e.g., general self‐efficacy, safety climate and organisational learning climate) and behavioural or performance indicators (e.g., the number of quality improvement projects in which a nurse has participated, the frequency of error or near‐miss reporting and participation in quality improvement training programmes). Such data would make it possible to examine more comprehensively the convergent, discriminant and criterion‐related validity of the Mandarin‐Quality Improvement Self‐Efficacy Inventory.

The model fit aims to ensure that the population model‐implied covariance matrix Σ (θ) fits the actual population covariance matrix Σ. However, given the unavailability of both Σ (θ) and Σ, assessing the model fit involves making the covariance matrix, as implied by the hypothesised model, denoted as, as close to the sample covariance matrix **S** as possible by minimising the discrepancy. The achieved minimum value of the discrepancy function in a sample of size *N*, denoted as, reflects the degree of lack of fit of the model to the sample data. The *H*_0_ is that the population discrepancy *F*_0_ between the hypothesised covariance matrix Σ (θ) and the actual population covariance matrix Σ is zero (i.e., *H*_0_: Σ = Σ (θ)). When *H*_0_ is true, that is, the model is precisely correct in the population, and any lack of fit to the sample data results arises only from sampling error, the test statistic (*N* −  1) follows a central *χ* ^2^ (df) distribution asymptotically with the degrees of freedom. A critical value test statistic is obtained from the central *χ* ^2^ (df) distribution based on the specified α level. The *p* value indicates the probability of obtaining a test statistic as extreme as that observed in the given sample, assuming that *H*_0_ holds in the population. If this probability falls below the α level, *H*_0_ is rejected, suggesting that the model does not fit exactly in the population. However, since the conclusions are drawn from observed sample statistics, two types of error can occur when testing statistical hypotheses: Type I error (*α* error), which involves incorrect rejection of a true model, and Type II error (β error), which involves the incorrect retention of a false model. Figure 4(a) presents the relationship between *H*_0_ (truth/false) and the test conclusions. In our study, we set *α* = 0.05, ensuring a low probability of Type I error. However, if the model is incorrect in the population and we test the *H*_0_ of exact fit, we may fail to reject *H*_0_ if we extract sample data from a good model fit or if the model contains a serious specification error, but with a small sample. Therefore, a power analysis is important for model testing. When *F*_0_ ≠ 0, the test statistic (*N* −  1) follows a noncentral *χ* ^2^ (df, λ) distribution with df and noncentrality parameter λ. The noncentrality parameter *λ* = (*N* − 1)*F*_0_ quantifies the degree of misspecification error. Therefore, the alternative hypothesis, *H*_1_, is equivalent to specifying an unacceptable degree of model misfit. Figure 4(b) illustrates how the α and β errors arise in the hypothesis testing. Since the scaling of *F*_0_ is difficult to deal with directly, the non‐centrality‐based model fit index, root mean square error of approximation, was used to express the population discrepancy. The probability of falsifying our two‐factor model when it was wrong to an extent corresponding to RMSEA = 0.08 at *α* = 0.05 was 96%, indicating that missing a model with clearly poor fit is unlikely. Therefore, we are confident that the two‐factor model provides an adequate description of the data (and implicit real‐world phenomena). At the same time, the power to detect a small deviation from a close fit corresponding to RMSEA = 0.05 was only 54%, suggesting limited sensitivity to very subtle misspecifications. Although the sample size met commonly recommended guidelines for EFA and CFA, the power analysis indicated that it was not sufficiently large to provide high power for detecting very subtle model misspecifications. Thus, while the combination of fit indices and power analysis supports the adequacy of the two‐factor solution as a parsimonious representation of the data, interpretations of ‘good fit’ should be made with the recognition that minor departures from an exact‐fitting model may have gone undetected. Future studies, especially those aiming to test measurement invariance across groups or to compare alternative complex models (e.g., higher order or bifactor structures), should therefore plan for larger sample sizes to ensure higher power for detecting close‐fit deviations. Our experience with the present power analysis will be used to guide more precise sample size planning in subsequent multicentre research on the Mandarin‐Quality Improvement Self‐Efficacy Inventory.

Figure 4(a) The relationship between the *H*_0_ (truth/false) and the test’s conclusions. (b) Central *χ* ^2^ (df) distribution (pink solid line), noncentral *χ* ^2^ (df, λ) distribution (green solid line), critical *χ* ^2^ value (dashed black line) and associated decision errors. The pink‐shaded area of the central distribution to the right of the critical value corresponds to the α error. The green‐shaded area of the noncentral distribution to the left of the critical value corresponds to the β error.

**Figure:**
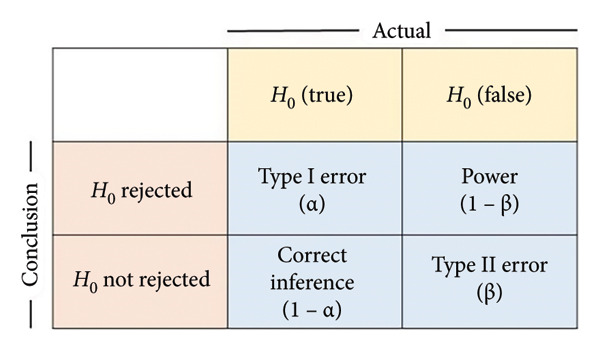
(a)

**Figure:**
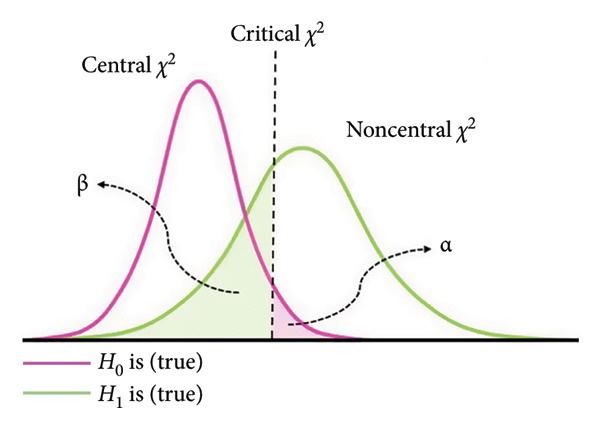
(b)

The four‐factor model should be rejected based on the selected *α* = 0.05 level. However, the *H*_0_ of the exact model fit might be overly optimistic in practice. Our compromise power analysis demonstrated that the four‐factor model was aligned closer to *H*_0_ than to *H*_1_. Thus, while the *H*_0_ of the exact model fit was violated, the four‐factor model could be considered valuable (an unacceptable degree of misfit is defined as a root mean square error of approximation of at least 0.1).

Considering cultural variations, we considered the Mandarin‐Quality Improvement Self‐Efficacy Inventory‐2 to be more appropriate for evaluating the quality improvement competence of Chinese nurses in Chinese healthcare settings. The Mandarin‐Quality Improvement Self‐Efficacy Inventory‐2 was sufficiently valid and reliable as a practical scale for measuring the quality improvement competency of Chinese nurses. Moreover, a novel finding in our study is that the original four‐factor structure of the Quality Improvement Self‐Efficacy Inventory was supported in China. The provision of high‐quality and safe patient care has become a major global concern. It is critical to conduct studies on quality improvement in international settings to generate a thorough understanding of how quality improvement competency is formed [22]. Thus, we believe that cross‐cultural research on quality improvement among nurses is promising and valuable. Researchers can use the Mandarin‐Quality Improvement Self‐Efficacy Inventory‐4 in future studies to examine the cross‐cultural measurement invariance of the Quality Improvement Self‐Efficacy Inventory, exploring whether the Quality Improvement Self‐Efficacy Inventory can be used for comparisons of nurses’ quality improvement competencies across different countries. In summary, we provide an open option that allows researchers to choose a suitable factor structure depending on the research objectives and needs of their study.

While the Mandarin‐Quality Improvement Self‐Efficacy Inventory did not add or delete any items, this methodological study addresses an issue that warrants further examination. It is common for the configuration of factors to vary when validating the scale across different populations. However, when faced with such results and ensuring acceptable reliability and validity, the question arises: Which option should be chosen? This subject is worthy of academic investigation. Since the judgement of model fit is subjective, it is imperative to present unquestionable statistical evidence and a convincing explanation for either retaining or rejecting the model. Stating differently, if proposing a new factor structure, it must possess significance to exist in reality and not just be based on the results of the data analysis. When conducting model fit testing, it is crucial to maintain a modest error probability (α and β) for making a sound choice between *H*_0_ and *H*_1_. Nonetheless, power analysis is often overlooked during model testing. Without confidence that the model adequately represents the data (and, by extension, real‐world phenomena), any proposed factor structure is meaningless. Correspondingly, the factor structure may emerge as more or less differentiated, and it would be imprudent to dismiss it lightly, particularly if it is grounded in theory. Instead of unilaterally discarding competing solutions to retain only a one‐factor structure, an open‐ended approach could be adopted. However, the premise is that the model must provide a phenomenon as close as possible to the real world, and each factor structure must be supported by statistical evidence and practical significance.

Therefore, establishing a factor structure is important for the development, translation and psychometric evaluation of measurement instruments. Some studies choose to replicate the original factor structure in the translation version of the measurement instruments, potentially hindering the discovery of more appropriate factor structures. Certain studies conduct new exploration and validation of the factor structure; however, does this imply that the original structure can be rationally abandoned when a new and better structure is established? In our view, the original factor structure should not be abandoned merely because a new superior alternative has emerged but rather because it is fundamentally flawed and cannot adequately represent real‐world phenomena. The establishment of a new factor structure indicates the discovery of a more appropriate model rather than the complete overturning of the original structure. Researchers typically conduct rigorous confirmatory studies to ensure statistical accuracy when establishing factor structures. Admittedly, if the studies had access to an infinitely large and perfectly clean dataset, the actual effects could be quantified; however, we were compelled to make do with a random sample. The results obtained from the sample may not accurately reflect the population’s reality, and random errors may lead to erroneous inferences. Therefore, it is important to reduce the overall rate of data inference error. In essence, we are ‘describing’ the underlying infinite datasets, and a mistake in judgement might have disastrous consequences, particularly when it comes to the measuring instrument that will be extensively utilised in a field. While α error and β error can never be entirely avoided, they should be minimised in practice. Unfortunately, the β error is rarely considered while making statistical decisions in factor structure studies.

In light of the aforementioned, two recommendations are proposed to enhance the rigour and applicability of factor structure research, particularly in the context of scale development, translation and psychometric evaluation. First, when adapting scales to new languages or cultural settings, it is essential to conduct a comprehensive and systematic re‐evaluation of factor structures, as the dynamic nature of cultural contexts may subtly influence how constructs are conceptualised, represented or measured. However, the emergence of a new model alone should not justify discarding the original structure unless empirical evidence demonstrates that the original model is statistically inadequate. Future nursing research will be a global collaborative effort focused on addressing the increasingly complex health challenges in a globalised world. Ensuring that the original factor structure demonstrates acceptable psychometric properties within a single cultural group is a fundamental prerequisite for conducting cross‐cultural measurement invariance testing and drawing valid conclusions. Consequently, the original factor structure should be systematically reported alongside the exploration of new factor structures, establishing a solid foundation for future, more comprehensive research on cross‐cultural measurement invariance. Second, incorporating power analysis into factor structure studies is essential to address the limitations of conventional validation methods, such as fit indices or exploratory factor analyses, which often overlook the risk of Type II error (β error). By incorporating power analysis to ensure that factor structures have sufficient statistical power to detect true underlying effects, the study reduces the risk of drawing false conclusions about spurious or misspecified structures arising from random error or sampling variability, ensures that observed results reflect substantive patterns in the data and enhances the statistical validity of research findings. Researchers should recognise that factor structure validation is not a rigid, one‐time task but an iterative process open to refinement and re‐evaluation. The crux of factor structure studies lies in achieving a balance between model flexibility and statistical rigour. This balance is crucial for advancing cross‐cultural psychometric research and ensuring the reliability and validity of adapted measurement instruments.

## 5. Limitations

First, this study was conducted in a single tertiary hospital in Tianjin, which may limit the generalisability of the findings to the broader population of nurses in China. Nurses in tertiary hospitals may receive more systematic training in quality improvement than those in primary care or rural settings, and their exposure to quality improvement projects, error reporting systems and safety culture initiatives may be higher. These contextual differences could inflate both their self‐efficacy and their familiarity with quality improvement terminology. As a result, the factor structure identified in this study may not fully capture how nurses working in other regions and at other levels of healthcare institutions conceptualise and experience quality improvement self‐efficacy. Future research should therefore conduct multicentre studies that include nurses from primary, secondary and tertiary institutions in both rural and urban areas, and use such multicentre samples to re‐examine the factor structure and to establish national norms for the Mandarin‐QISEI. Second, although an expert review and cognitive interviews were used in this study to qualitatively evaluate item relevance, clarity and cultural appropriateness, we did not compute quantitative content validity indices (CVIs) such as the item‐level and scale‐level CVI (I‐CVI and S‐CVI). This absence of formal CVI limits the strength of our content validity evidence. Another methodological limitation concerns the model modifications applied in the confirmatory factor analysis. The good fit of the two‐factor model was achieved after allowing a limited number of correlated residuals between conceptually overlapping items, based on MI. These modifications are partially data‐driven and may capitalise on sample‐specific characteristics. Therefore, the stability and generalisability of the model, including the need for residual correlations, should be examined in independent multicentre samples. In future studies, we recommend first testing the prespecified model without additional modifications and only introducing correlated residuals when there is a strong a priori theoretical justification. Fourth, future studies could explore and identify cut‐off points for the Chinese version, which could help further increase the practical value of the tool. Furthermore, while our study supports the original four‐factor structure of the Quality Improvement Self‐Efficacy Inventory in China, the validation of cross‐cultural measurement invariance could not be completed within the scope of this study. Conducting multigroup confirmatory factor analysis (MGCFA) to assess whether the factor structure holds equivalently across different cultural groups requires data collected from multiple countries or cultural contexts simultaneously, while our study only included a Chinese sample and lacked data from other countries or cultures. Advancing international research is a common measure that can be used across countries and cultures. Hence, an international, cross‐sectional, descriptive study should be conducted in the future to test the cross‐cultural measurement invariance of the Quality Improvement Self‐Efficacy Inventory.

## 6. Conclusions

The present study completed the translation of the original version of the Quality Improvement Self‐Efficacy Inventory from English to Mandarin and reported the psychometric properties of the Mandarin‐Quality Improvement Self‐Efficacy Inventory among Chinese nurses for the first time. We made an open option for the factor structure of the Mandarin‐Quality Improvement Self‐Efficacy Inventory. The Mandarin‐Quality Improvement Self‐Efficacy Inventory with the new two‐factor structure (Mandarin‐Quality Improvement Self‐Efficacy Inventory‐2) can be specifically adapted to gauge Chinese nurses’ quality improvement knowledge and skills. The Mandarin‐Quality Improvement Self‐Efficacy Inventory with the original four‐factor structure (Mandarin‐Quality Improvement Self‐Efficacy Inventory‐4) can be used as an instrument in future studies examining the cross‐cultural measurement invariance of the Quality Improvement Self‐Efficacy Inventory. In summary, the Mandarin‐Quality Improvement Self‐Efficacy Inventory served as a unique, relatively stable and brief scale with excellent reliability and validity. This methodological study proposes two key recommendations for future research in the development, translation and psychometric evaluation of measurement instruments: first, recommending a comprehensive re‐examination of the factor structure and rigorous statistical validation of both the original and newly derived structures in scale translation studies; and second, the incorporation of power analysis to enhance the statistical rigour of factor structure evaluation and reduce the risk of supporting spurious or misspecified structures. These recommendations highlight the importance of balancing statistical rigour with practical applicability, offering a methodological reference for factor structure research in the development, translation and psychometric evaluation of measurement instruments [23, 24].

## Disclosure

All the authors reviewed and approved the final manuscript and agreed to its submission.

## Conflicts of Interest

The authors declare no conflicts of interest.

## Author Contributions

All authors listed on the manuscript have made substantial contributions to this work. Nuoyan Zhang contributed to the methodology and formal analysis and wrote the original draft. Xiaoxiao Zhang and Zhaonan Wang contributed to the investigation, formal analysis, validation and writing–review and editing. Yuting Feng, Hangjie Lian, Haoquan Han, Xinyu Bai, and Yaqi Huang contributed to the investigation, data curation and visualisation. Qi Lu, Yulu Wang, and Yue Zhao contributed to the conceptualisation, supervision and Writing–review and editing. Nuoyan Zhang, Xiaoxiao Zhang and Zhaonan Wang made equal contributions to this manuscript and should be regarded as co‐first authors.

## Funding

This work was supported by the National Natural Science Foundation of China (72274134, 72574164, 72404208, 72204186, and 71974142).

## Data Availability

The data that support the findings of this study are available from the corresponding author upon reasonable request.
