# Correction to “Psychometric Properties of the Mandarin Version of the Quality Improvement Self‐Efficacy Inventory Among the Nurses: A Methodological Study”

**DOI:** 10.1155/jonm/9842073

**Published:** 2026-05-17

N. Zhang, X. Zhang, Z. Wang, et al., “Psychometric Properties of the Mandarin Version of the Quality Improvement Self‐Efficacy Inventory Among the Nurses: A Methodological Study,” *Journal of Nursing Management* 2026 (2026): 4425503, https://doi.org/10.1155/jonm/4425503.

In the article titled “Psychometric Properties of the Mandarin Version of the Quality Improvement Self‐Efficacy Inventory Among the Nurses: A Methodological Study,” an affiliation was omitted for the author “Xiaoxiao Zhang” in error. The corrected author list and affiliation list should be as follows:

Nuoyan Zhang^1,2^, Xiaoxiao Zhang^1,2,3^, Zhaonan Wang^4^, Yuting Feng^1,2^, Hangjie Lian^1,2^, Haoquan Han^1,2^, Xinyu Bai^1,2^, Yaqi Huang^5^, Yulu Wang^1,2^, Yue Zhao^1,2,6^, Qi Lu^1,2,6^

^1^Intelligent Nursing Research Center, Tianjin Medical University, Tianjin, China

^2^School of Nursing, Tianjin Medical University, Tianjin, China

^3^Chinese Academy of Medical Sciences and Peking Union Medical College, Institute of Hematology and Blood Diseases Hospital, Tianjin, China

^4^Tianjin Medical University General Hospital, Tianjin, China

^5^School of Nursing, The Hong Kong Polytech University, Hong Kong, China

^6^Joint Research Centre for Primary Health Care, The Hong Kong Polytechnic University, Hong Kong, China

We apologize for this error.

